# Completeness and Factors Affecting Community Workers’ Reporting of Births and Deaths in the Countrywide Mortality Surveillance for Action in Mozambique

**DOI:** 10.4269/ajtmh.22-0537

**Published:** 2023-04-10

**Authors:** Almamy M. Kante, Azarias Mulungo, Mussagy Ibraimo, Aveika Akum, Nordino Titus, Antonio Adriano, Fred Van Dyk, Ivalda Macicame, Robert E. Black, Agbessi Amouzou, the COMSA-Mozambique Study Working Group

**Affiliations:** 1 Johns Hopkins Bloomberg School of Public Health, Baltimore, Maryland;; 2 Instituto Nacional de Saude, Maputo, Mozambique;; 3 Instituto Nacional de Estatistica, Maputo, Mozambique

## Abstract

Since March 2018, the Countrywide Mortality Surveillance for Action project, implemented as a national sample registration system by the Mozambique Instituto Nacional de Saude and the Instituto Nacional de Estatistica in 700 geographic clusters randomly distributed across the 11 provinces, has trained and deployed community surveillance agents (CSAs) to report births and deaths in each cluster prospectively. An independent, retrospective data collection was conducted to assess the completeness of surveillance data. Record linkage procedures were used to match households and vital events reported in the two data sources. We calculated birth and death reporting rates and used a regression model to determine factors associated with the likelihood of vital events being reported by the CSAs. Between March 2018 and December 2019, CSAs reported 54% of births (8,787/16,421) and 45% of deaths (1,726/3,867). Births of smaller cluster sizes (< 1,000 people) were more likely to be reported (adjusted odds ratio [aOR] = 1.45; 95% CI = 1.15–1.83) compared with those of larger cluster sizes (> 1,500 people). Deaths of rural clusters were more likely to be reported (aOR = 1.41; 95% CI = 1.07–1.85) than those of urban clusters. Adult deaths were more likely to be reported (aOR = 1.49; 95% CI = 1.10–2.02) than child deaths. Our findings suggest that a fully functioning sample vital registration system must adopt a dual system with high-quality surveys or other ways to estimate underregistration periodically, consider a smaller cluster size manageable by a community worker, and pay special attention to urban clusters as underreporting is larger.

## INTRODUCTION

Timely and reliable data on mortality trends and causes of death are fundamental to monitoring population health and informing public health policy.^1^^,^^2^ Functioning civil registration and vital statistics (CRVS) systems can provide governments with important information about births, deaths, and causes of death.^3^ However, in low- and middle-income countries (LMICs), CRVS systems are incomplete and therefore unable to produce reliable vital statistics.^4^ In sub-Saharan Africa, less than half of births and one-fifth of deaths are registered by official civil registration systems.^5^ Mortality statistics from the health service are also incomplete due to the limited utilization of facility services, with many deaths occurring outside of formal health systems.^3^ For example, in 29 sub-Saharan African countries, only 22% of births occurred in a health facility,^6^ and half of under-five deaths occurred at home.^7^ Most LMICs rely on population censuses and household surveys to produce mortality estimates, which are conducted every 10 and 3–5 years, respectively.^8^

A strong community-based national system for timely and accurate recording of vital events is urgently needed in LMICs to support the monitoring and evaluation of health programs and to assess progress toward the Sustainable Development Goals (SDGs).^8^ A sample vital registration system (SRS), consisting of a nationally representative sample of communities to continuously track population, births, deaths, and causes of death, can complement other sources by producing accurate and timely mortality estimates.^9^^,^^10^ However, an SRS is currently being implemented in a few LMICs, including China since the 1950s,^11^^,^^12^ India since the 1970s,^13^ Bangladesh since the 1980s,^14^ Indonesia since 2014,^15^^–^^17^ Tanzania^18^ and Zambia since 2010 for variable periods, and Sierra Leone since 2018.^19^

A scoping review of the reporting of community-based maternal and child deaths showed that only 4 out of 43 studies have reported about accuracy and completeness of death data.^20^

The validity and completeness of the reporting of community-based births and deaths have been studied in many settings.^11^^,^^17^^,^^21^^–^^27^ Underreporting of community births and deaths varies across studies. In the context of an SRS, the underreporting death rates were about 13% in China^11^^,^^24^^–^^26^ and 50% in Indonesia.^17^ A multi-country study on the validation of community vital events conducted in sub-Saharan Africa found that community workers have reported about 90% of births and under-five deaths in Mali, one-half of births and deaths in Malawi, and only one-third of births and deaths in Ethiopia.^21^^–^^23^ Nevertheless, little is known about factors affecting the underreporting of vital events by community workers.

Mozambique is a developing country in southern Africa that is among the few countries in Africa that reached the Millennium Development Goals for child mortality. To continue its health progress, the government needs a robust national data system to monitor progress toward SDGs.^28^ Mozambique launched an SRS in 2017, known as the Countrywide Mortality Surveillance for Action (COMSA), that covers 700 communities randomly distributed across the 11 provinces.^9^ In 2018, COMSA selected, trained, and deployed 700 community surveillance agents (CSAs). As of December 2020, the CSAs have registered about 800,000 people, 40,000 births, and 10,000 deaths.^29^ This paper describes the completeness of birth and death reporting by CSAs and individual-, household-, and community-level factors affecting the reporting of community vital events. The understanding of these factors will help fine-tune community level reporting and improve the completeness of reporting.

## MATERIALS AND METHODS

COMSA used a phased implementation with data collection starting in March 2018 in five northern provinces (Cabo Delgado, Zambezia, Tete,^30^ Nampula, and Sofala). This first phase included 422 communities. In October 2018, the data collection began in the second phase in the remaining provinces, including 278 communities. Using the crude birth and death rates from the 2017 Mozambique census data,^30^ our preliminary calculations suggested that CSAs may have been missing at least half of births or deaths. During the study design, an update of the population was planned during the third year of the project implementation.^29^ Therefore, the project leaders decided to conduct this assessment to verify the completeness and accuracy of the surveillance data.

The assessment survey was an independent, retrospective data collection activity in which all the families in the COMSA communities were revisited and questions were asked about births and deaths in their families during the reference period of 2018–2020. Using shortened versions of the surveillance data collection tools, the following information was collected: 1) household listing, including name, age, sex, and relationship to head of household; 2) births, including parents’ names and child’s name, birth status (alive or dead), sex, date of birth, and relationship to head of household; and 3) deaths, including parents’ names and deceased’s name, sex, date of birth and death, and relationship to head of household.

The data collection team included the COMSA verbal and social autopsy (VASA) interviewers acting as supervisors, plus an additional 120 interviewers recruited and trained for the assessment. A standardized, cascade training was conducted, with a 5-day training of trainers, then pilot testing, then province-level training of interviewers, each of which lasted 7 days. As in the COMSA surveillance, interviewers collected data on tablet computers equipped with Open Data Kit software.^31^^,^^32^ Tablets were equipped with Geopaparazzi software, allowing interviewers to identify the already digitized clusters’ boundaries and to work within boundaries.^33^ An update of cluster boundaries and household lists was conducted in 2018 prior to CSA deployment, allowing the digitization of each cluster map with clear boundaries.^29^ The cluster maps created after the cartography were also uploaded to each tablet to support the fieldwork. Finally, the complete list of households, including their members and vital events (births and deaths) reported by CSAs since the project’s onset, were downloaded onto each tablet.

Each data collection team was composed of one supervisor and three data collectors. The number of teams deployed varied by the number of clusters in the province, as presented in Table 1.

**Table 1:** Number of COMSA clusters and assessment data collection per province

Province	Number of COMSA clusters	Number of clusters assessed	Number of data collection teams	Data collection
Niassa	40	39	3	September–October 2020
Cabo Delgado	113	75	6	September–October 2020
Nampula	53	52	4	September–October 2020
Zambezia	118	118	8	December 2019–December 2020
Tete	106	106	6	December 2019–December 2020
Manica	85	84	6	September–October 2020
Sofala	29	29	3	September–October 2020
Inhambane	49	49	4	March–September 2020
Gaza	36	36	3	March–September 2020
Maputo Province	36	36	3	November 2019–September 2020
Maputo City	36	36	3	September–October 2020

COMSA = Countrywide Mortality Surveillance for Action.

The data collection started in November 2019 in Maputo province as a pilot phase and then extended to Tete and Zambezia provinces in December 2019. Data collection was expected to last about 2 months in each province but was delayed by heavy rains and then halted temporarily by the COVID-19 pandemic that hit the country in March 2020. As a result, data collection was later staggered by province and conducted between August and December 2020. Forty-one clusters were not assessed due to political instability, primarily in Cabo Delgado (*n* = 38) and to the heavy rainy season or difficult access (clusters not reachable) in Niassa (*n* = 1), Nampula (*n* = 1), and Manica (*n* = 1).

In the field, each interviewer was tasked to confirm the cluster boundaries and then to visit all households within each cluster. The GPS coordinates were taken for each household. The interview was conducted with the head of household or another adult household member who could provide accurate information on family members and births and deaths in the reference period; details about household-level data collection are explained in detail elsewhere.^29^ Births and deaths were documented since January 2018 for phase 1 provinces and since June 2018 for phase 2 provinces. Extending the events’ recollection to commence from a time point 3 months prior to the start of the routine CSA data reporting was intended to increase the likelihood that all births and deaths were captured. CSAs and community leaders assisted the data collectors.

The assessment data were matched with the routine surveillance data from the CSAs to ascertain the accuracy and completeness of the surveillance data. Data errors could come from three sources: 1) CSAs not collecting data from all the households in their assigned clusters, 2) CSAs visiting households but missing some births or deaths within those households, and 3) CSAs collecting data from households that were outside the boundaries of their assigned clusters.

Two levels of matching procedures were used for this activity. First, the list of households, including household identification number and names of the head and other household members, was provided to the interviewers to find the households on the ground. The list of births and deaths reported by the CSA prior to the assessment was also provided, and assessment interviewers were tasked with matching all households and reported vital events. Second, during the data processing, all households listed during the assessment were matched against the CSA household listing (surveillance data). This matching was done based on variables such as cluster identification (cluster code and CSA identification number provided by the project), household identification (number and name of the head of household), and identifying information about the vital events, such as year of birth or death, sex of the baby or the deceased, age of the deceased, and names of the parents. Three levels of data assessment were used during the field activities to ensure quality. The first level relied on controls and checks implemented in the electronic data reporting software. The second level assessment was conducted by the COMSA provincial-level teams. The COMSA provincial coordinators and VASA data collectors were posted in the field during this activity to supervise the fieldwork. They were tasked with revisiting a random sample of reported events to verify the accuracy of the information reported by interviewers. This verification scheme was set up in the data reporting system daily. The sample of events confirmed was accessible to and verifiable by the COMSA investigators. This assessment, however, could confirm the accuracy of events reported. The third level of quality assurance was the collection monitoring. We developed statistical analysis to compare the completeness of events reporting and of information collected from the surveillance and the assessment and from the assessment and the supervision (re-interview of selected cases by COMSA provincial teams). The results helped to make implementation corrections while interviewers were still in the field.

Data were cleaned, and variables were standardized before the linkage.^34^ Data cleaning included the removal of punctuation marks, accents, repeated blanks, and prepositions; conversion of letters to uppercase; removal of numbers from variables intended to be exclusively composed of letters and vice versa; and standardization of date formats. Subsequently, new variables were generated from standardized names (baby, deceased, and mother) by parsing (separation of fragments into the first name, second name, and so forth) and by keeping the first three variable names created. More than 99% of people have up to three names in this setting.

Record linkage uses deterministic or probabilistic approaches.^35^^–^^38^ Experience from Brazil (a Portuguese-speaking country like Mozambique)^39^ showed a high level of correlation for pair classification, but the probabilistic linkage retrieved links unidentified by the deterministic linkage. In the deterministic approach, a link is made if all fields agree. This approach does not fit the COMSA dataset because of spelling errors in the names and approximate dates of birth and death. Instead, we used a probabilistic approach that generates multiple possible matches and associates a probability of accuracy with each match.^35^^,^^36^ We used a minimum matching score of 0.6 as the default in Stata statistical software (version 16.0) to increase matching rates even if it required extra steps to clean up the additional pairs (duplicates).

**Figure 1.**
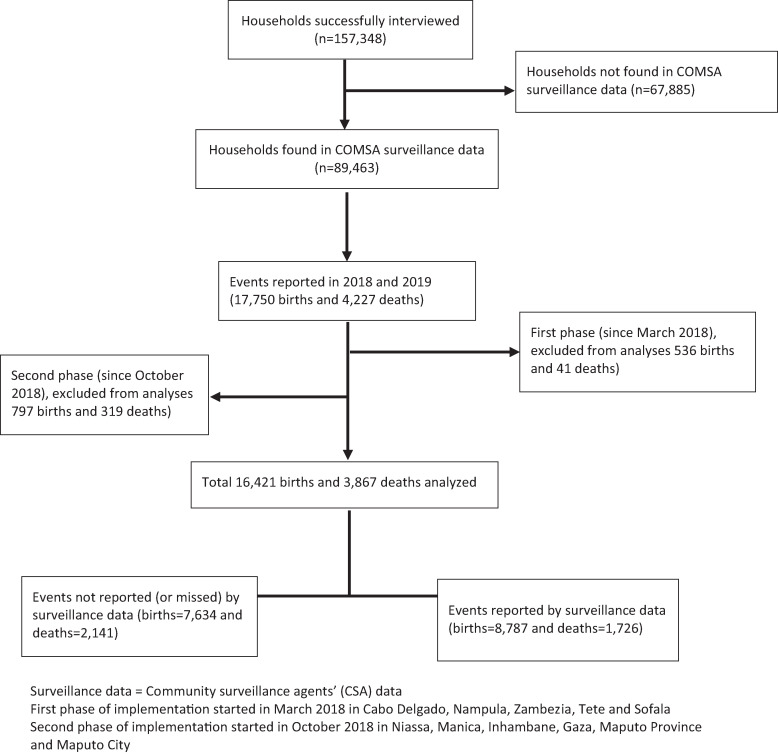
Flow diagram of data obtained from the Countrywide Mortality Surveillance for Action (COMSA) assessment survey in 2020.

Of the 159,854 households visited during the assessment, 98.4% (*n* = 157,348) participated in the study; 89,463 (56.9%) households were reported by the CSA, and 67,885 (43.1%) households were missed (not reported) by the CSA because many of them have been working outside their clusters’ boundaries. We restricted the analysis to households found in both datasets (*n* = 89,463). Therefore, we assumed that the findings would be similar in areas not covered by the CSAs. The assessment data collectors reported 17,750 births and 4,227 deaths in 89,463 households between January 2018 and December 2019. Events before the start of the COMSA data collection were removed from the analysis (1,333 births and 360 deaths). The total sample included in this study is 16,417 births and 3,867 deaths between March 2018 and December 2019 (Figure 1).

We initially assessed the completeness of surveillance data (births and deaths reported by the CSAs) by using the assessment dataset as the reference. We then developed analyses to define the characteristics of vital events that were likely to be reported by the CSAs.

We conducted hierarchical multivariate analyses of the likelihood of the CSAs reporting a vital event (birth or death).^40^^,^^41^ Individuals formed level 1 of the analysis and were grouped by cluster or community (level 2). A regression model was constructed to test the effect of individual and community-level covariates, and those that improved the model, as determined by statistical significance testing, were retained (*P* < 0.05). Our final multilevel model was as follows:

$$\log\left[\mathrm{Pr}\left(Y_{ij}=1\right)\right]=\beta 0+\sum_{f=1}^{F}\beta fxf_{ij}+\sum_{h=1}^{H}\beta_{h}\delta_{hj}+\mu_{oj}$$

where *Y_ij_* is a categorical variable coded 1 if birth or death of *i*^th^ subject (baby or deceased) within the *j*^th^ cluster is reported in the surveillance data and 0 otherwise, ***x**f_ij_* denotes *f* predictors measured on this subject (sex, age), **δ***_hj_* denotes *h* predictors measured on the *j*^th^ cluster (size; location; and CSA characteristics such as age, sex, marital status, education level), and **β** is vector of coefficients. The term μ*_oj_*, assumed to be normally distributed, refers to cluster-level random effects.

The study protocol was reviewed and approved by the Johns Hopkins Bloomberg School of Public Health’s Institutional Review Board (IRB#7867) and the National Health Bioethics Committee of Mozambique (REF 608/CNBS/17). The data collectors obtained informed consent before the data collection. The consent forms were translated into Portuguese and the local languages in each province.

## RESULTS

### Population characteristics.

The characteristics of the study population are presented in Table 2. Of the 16,421 births reported during the assessment, 98% of the neonates were born alive, with equal distribution among girls and boys. Approximately 70% of babies were the biological children of the head of the household. Less than 30% of births reported were in clusters, with < 1,000 people and 71.8% in rural clusters.

**Table 2:** Proportional distribution of characteristics of the study population and vital events (births and deaths), and the proportion of events in that category reported by the community surveillance agents

Characteristics	Categories	Births			Deaths		
		Assessment sample (%)	Reported by CSAs (%)	*n* *	Assessment sample (%)	Reported by CSAs (%)	*n* *
Status of the birth	Born alive	98.2	53.6	16,124	–	–	–
	Born dead (stillbirth)	1.8	48.8	297	–	–	–
Age of deceased, years	< 1	–	–	–	14.1	43.8	543
	1–4	–	–	–	13.7	41.0	530
	5–14	–	–	–	7.4	42.0	286
	15–49	–	–	–	30.7	42.2	1,196
	≥ 50	–	–	–	31.8	49.4	1,224
Sex of baby/deceased	Female	49.8	54.4	8,029	45.0	44.0	1,740
	Male	50.2	52.8	8,075	55.0	45.2	2,125
Relationship to head of household	Head or partner	–	–	–	29.7	51.7	1,150
	Biological child	70.5	57.0	11,545	39.3	44.4	1,519
	Grandchild/close family	29.5	44.9	4,579	31.0	38.2	1,198
Year of event	2018	27.7	58.9	4,556	23.5	43.9	907
	2019	72.3	51.4	11,865	76.5	44.9	2,960
Sex of the head of household	Female	21.6	51.0	3,479	34.8	47.4	1,347
	Male	78.2	54.4	12,602	62.7	43.0	2,424
Age group of head of household, years	< 30	27.4	57.2	4,437	14.0	45.8	542
	30–49	50.6	55.0	8,152	41.1	43.0	1,590
	≥ 50	20.9	45.5	3,370	40.9	45.7	1,581
Household has a phone	Yes	47.2	54.4	7,596	52.5	44.0	2,031
	No	52.8	52.9	8,528	47.5	45.3	1,836
Household structure (age/sex distribution) (age in years)	One-third < 15	18.7	50.1	2,963	41.5	45.5	1,606
	One-third 15–49	31.9	54.4	5,175	37.7	46.4	1,456
	One-third ≥ 50	99.2	53.7	15,993	84.9	43.9	3,282
	One-third female, 15–49	99.2	53.7	16,002	97.6	44.9	3,775
Household size, *n*	< 5	20.4	57.7	3,272	23.6	45.7	914
	5–7	43.7	54.8	7,065	40.6	44.8	1,571
	≥ 8	35.8	49.9	5,787	35.7	43.7	1,382
Household deaths, *n*	1	99.1	53.6	15,988	82.8	45.4	3,201
	≥ 2	0.9	2.2	136	17.2	41.0	666
Household births, *n*	1	88.6	54.3	14,277	96.6	44.6	3,734
	≥ 2	11.4	48.5	1,847	3.4	44.4	133
Cluster size (no. of households)	< 200	25.9	60.4	4,175	21.4	53.9	828
	200–299	48.1	54.0	7,762	49.2	44.8	1,901
	≥ 300	26.0	46.1	4,187	29.4	37.7	1,138
Cluster size (no. of people)	< 1,000	27.0	59.2	4,344	22.2	54.1	860
	1,000–1,499	44.3	54.2	7,160	44.1	45.2	1,705
	≥ 1,500	28.7	47.5	4,620	33.7	37.7	1,302
Cluster deaths, *n*	< 10	33.8	56.5	5,467	18.0	48.3	696
	10–19	43.2	55.8	6,974	47.7	50.2	1,846
	≥ 20	23.0	45.2	3,683	34.3	35.0	1,325
Cluster births, *n*	< 30	10.9	54.8	1,794	18.0	48.3	696
	30–59	43.7	55.6	6,995	47.7	50.2	1,846
	≥60	45.4	51.4	7,335	34.3	35.0	1,325
Cluster residence area	Urban	28.2	46.8	4,543	35.9	37.8	1,388
	Rural	71.8	56.3	11,581	64.1	48.5	2,479
Province of residence	Niassa	6.1	55.0	978	4.3	27.3	165
	Cabo Delgado	9.1	46.0	1,458	10.1	33.7	389
	Nampula	6.2	57.2	999	7.2	41.6	279
	Zambezia	17.1	45.0	2,757	15.5	42.5	600
	Tete	20.0	57.4	3,241	12.3	52.0	477
	Manica	11.7	67.2	1,878	10.8	54.7	417
	Sofala	5.3	60.1	846	7.0	31.0	271
	Inhambane	7.2	54.7	1,160	9.9	45.8	384
	Gaza	6.8	52.2	1,090	8.0	59.9	309
	Maputo Provincia	5.4	50.9	877	7.2	42.8	278
	Maputo Cidade	5.2	40.7	840	7.7	46.6	298
Phase of implementation	First Phase (March 2018)	57.7	52.1	9,301	52.1	41.4	2,016
	Second Phase (October 2018)	42.3	55.6	6,823	47.9	48.2	1,851
Total	Percent	100.0	53.5	100	100.0	44.6	100
	*n* *	16,421	8,787	16,421	3,867	1,726	3,867

* Total number of births or deaths reported in the assessment dataset.

Source: COMSA, 2021.

**Table 3:** Characteristics of CSA

Characteristics	Categories	*n* *	%
Sex	Male	459	73.4
	Female	167	26.6
Age group,† years	< 30	252	40.4
	30–39	206	32.6
	≥ 40	168	27.1
Education level	Primary	159	26.1
	Secondary	437	69.2
	Higher	30	4.7
Marital status	Married/life partner	438	70.3
	Single/divorced/widowed	188	29.7
Live in the COMSA cluster	Yes	288	46.7
	No	338	53.3
Time to reach the nearest household (minutes)	0 (CSA lives in the community)	288	46.7
	< 15	116	18.4
	15–45	98	15.3
	≥ 45	124	19.6
Role in the community	CSA only	288	46.7
	APE	77	12.5
	Chief/leader/deputy	50	8.1
	Activist (member/secretary)	128	20.3
	Other	83	12.5
Total		626	100

APE = agente polivalente elementare; COMSA = Countrywide Mortality Surveillance for Action; CSA = community surveillance agent.

* Total number of CSAs in the assessment dataset.

† Mean age: 34.6 years (SD = 10.9).

Source: COMSA, 2021.

**Table 4:** Multilevel logistic regression models of the odds of reporting vital events (births and deaths) by the community surveillance agents

Characteristics	Categories	Births reported		Deaths reported	
		OR	95% CI	OR	95% CI
Status of the birth	Born dead (stillbirth)	Ref.		–	–
	Born alive	1.29*	1.01–1.67	–	–
Sex of baby/deceased	Male	Ref.		Ref.	
	Female	1.08*	1.01–1.16	1.05	0.90–1.22
Age of deceased, years	< 1	–	–	Ref.	
	1–4	–	–	0.87	0.66–1.15
	5–14	–	–	0.98	0.70–1.36
	15–49	–	–	1.14	0.88–1.49
	≥ 50	–	–	1.49†	1.10–2.02
Relationship to head of household	Close family	Ref.		Ref.	
	Biological child	1.48‡	1.34–1.64	1.54‡	1.24–1.90
	Head or partner	–	–	1.69‡	1.38–2.07
Household head’s sex	Male	Ref.		Ref.	
	Female	0.98	0.90–1.08	1.07	0.90–1.26
Household head’s age group, years	≥ 50	Ref.		Ref.	
	< 30	1.23†	1.08–1.39	1.10	0.87–1.40
	30–49	1.19†	1.07–1.32	1.06	0.90–1.26
Cluster size (no. of people)	≥ 1,500	Ref.		Ref.	
	< 1,000	1.45†	1.15–1.83	1.83‡	1.32–2.55
	1,000–1,499	1.16	0.95–1.42	1.17	0.90–1.55
Cluster residence area	Urban	Ref.		Ref.	
	Rural	1.27*	1.04–1.55	1.41*	1.07–1.85
CSA age group, years	< 25	Ref.		Ref.	
	25–49	1.14	0.91–1.44	1.78‡	1.29–2.46
	≥ 50	1.07	0.77–1.48	1.77*	1.12–2.79
CSA sex	Male	Ref.		Ref.	
	Female	0.94	0.77–1.15	0.80	0.64–1.05
CSA education level	Secondary and higher	Ref.		Ref.	
	Primary	1.22*	1.01–1.48	1.35*	1.03–1.76
CSA marital status	Single/divorced/widowed	Ref.		Ref.	
	Married/life partner	1.06	0.89–1.28	1.35*	1.041.74
CSA lives in/outside	Outside the community	Ref.		Ref.	
	In the community	1.25*	1.05–1.49	1.28*	1.02–1.60
CSA role in the community	CSA	Ref.		Ref.	
	APE	0.82	0.61–1.11	0.80	0.51–1.23
	Other	0.98	0.70–1.22	0.90	0.67–1.21
Province of residence	Niassa	2.13‡	1.45–3.15	0.54*	0.29–0.98
	Cabo Delgado	1.15	0.86–1.60	0.61*	0.39–0.94
	Nampula	2.07‡	1.49–2.92	0.90	0.56–1.44
	Zambezia	Ref.		Ref.	
	Tete	1.83‡	1.40–2.39	1.30	0.87–1.95
	Manica	3.64‡	2.83–5.49	1.86*	1.15–2.98
	Sofala	2.79‡	1.94–4.22	0.77	0.46–1.29
	Inhambane	2.24‡	1.61–3.43	1.42	0.84–2.41
	Gaza	2.51‡	1.77–4.20	3.04‡	1.69–5.46
	Maputo Provincia	2.12‡	1.51–3.41	1.29	0.74–2.21
	Maputo Cidade	2.01†	1.39–3.31	2.68†	1.51–4.78
Total, *n*§		16,421		3,867	
Model statistics	Constant	0.11	0.06–0.22	0.16	0.13–0.22
	SD (constant)	0.78	0.72–0.85	0.82	0.71–0.95
	Log-likelihood	−10,399.9		−2,413.98	
	Wald χ^2^ (df)	311.60 (34)		179.08 (37)	
	*P* > χ^2^	0.000	–	0.000	–
	Likelihood ratio test vs. logistic model, χ^2^ (df)	Prob > = 1,098.72 (1) = 0000		Prob > = 131.91 (1) = 0000	

APE = agente polivalente elementare; COMSA = Countrywide Mortality Surveillance for Action; CSA = community surveillance agent; OR = odds ratio. Since 1978, Mozambique has a national cadre of CHWs, known as APEs, to conduct health promotion activities (80% of their time) and provide integrated community case management for malaria, pneumonia, and diarrhea and register all births and deaths in their communities.^56^ In 2017 there were about 3,380 APEs in the country, mainly located in rural areas.^50^ The MISAU has allowed the project to employ APEs in the COMSA clusters (*n* = 77) where they were already working to report community vital events using the COMSA tools.

* Statistically significant at *P* < 0.05.

† Statistically significant at *P* < 0.01.

‡ Statistically significant at *P* < 0.001.

§ Total number of births or deaths reported in the assessment dataset.

Of the 3,867 deaths reported during the assessment, about 14% were infants, 14% were children 1–59 months old, and > 62% were 15 years old and above. More than half (55%) of subjects who died were male. Approximately 30% of deaths were heads of the household or their partners, and 39% were their biological children. Twenty-two percent of deceased subjects lived in clusters with < 1,000 people, and 64% lived in rural clusters.

Almost three-quarters (73%) of CSAs were male, and 40% were < 30 years old. Most CSAs attained secondary level education (70%), and a few reached a higher level of education (5%). Approximately 71% of CSAs were married or living with a life partner. Nearly half of CSAs (47%) lived in the cluster where they worked, 18% lived < 15 minutes walking distance to the community, and 35% lived ≥ 15 minutes walking distance to the community. Nearly half (47%) of CSAs did not have another community role, 12% were also agentes polivalentes elementares (APEs),^1^ 8% were community chiefs, and 20% were activists or members of a community organization (Table 3).

Between March 2018 and December 2019, the assessment recorded 16,421 births (including 297 stillbirths) and 3,867 deaths in the 89,463 households found in the CSA reports. Of these, 53.5% and 44.6% were reported by the CSA, respectively.

Over half of babies born alive (54%) were reported by the CSA, compared with 49% of babies born dead (stillbirths). Among the live births, 54% of girls were reported, compared with 53% of boys. Biological children of the household head were more likely to be reported than births from other household members (57% versus 45%). Over half of the babies in a household headed by a male (54%) or by a person < 30 years old (57%) were reported, compared with babies in a household headed by a female (51%) or by an adult ≥ 50 years old (46%). About 60% of babies in smaller clusters (< 200 households or 1,000 people) were reported, compared with 47% of babies in larger clusters (> 300 households or 1,500 people). More than half (56%) of babies of rural clusters were reported, compared with 47% of babies of urban clusters. The level of reporting also varied substantially by province. About 45% of babies in Zambezia province were reported, compared with 67% in Manica.

Approximately 43% of deaths of children < 1 year of age were reported, compared with 49% of adult deaths among those ≥ 50 years old. Male and female deaths were similarly reported at 45%. More than half (52%) of deaths of heads of household and their partners were reported, compared with 38% of deaths of other family members. About 54% of deaths in smaller clusters (< 200 households or 1,000 people) were reported, compared with 38% of deaths in larger clusters (> 300 households or 1,500 people). Almost half (49%) of deaths in rural clusters were reported, compared with 38% of deaths in urban clusters. About 27% of deaths in Niassa were reported, compared with 60% of deaths in Gaza.

Overall, we noted a difference of 9 percentage points (pp) in the levels of birth and death underreporting. There were also little variations across the characteristics of babies and deceased. However, considerable differences were found at the provincial level in Sofala (29 pp) and Niassa (28 pp).

### Factors associated with the likelihood of capturing births by CSAs.

Among individual-level factors considered, babies born alive were more likely to be reported than stillbirths (odds ratio [OR] = 1.29; 95% CI = 1.01–1.66). Among live births, female babies were more likely to be reported than male babies (adjusted OR [aOR] = 1.08; 95% CI = 1.01–1.16). Biological children of the head of household were also more likely to be reported than babies of other household members (aOR = 1.48; 95% CI = 1.34–1.64). Births in households headed by younger people were more likely to be reported than births in households headed by older people (for those < 30 years old, aOR = 1.23; 95% CI = 1.08–1.39).

Among cluster- and CSA-level factors, births in smaller clusters were more likely to be reported than births in larger clusters (for < 1,000 people: aOR = 1.45; 95% CI = 1.15–1.83). Births in rural clusters were more likely to be reported than births in urban clusters (aOR = 1.27; 95% CI = 1.04–1.55).

Births in clusters monitored by less-educated CSAs were more likely to be reported than births in clusters monitored by higher-educated CSAs (aOR = 1.22; 95% CI = 1.01–1.48). Births in clusters where the CSAs live in the cluster were more likely to be reported than births in clusters where the CSAs live outside (aOR = 1.25; 95% CI = 1.05–1.49). Births in nearly all provinces were more likely to be reported than births in Zambezia (for Manica: aOR = 3.64; 95% CI = 2.83–5.49) (Table 4).

In contrast, characteristics of the household (e.g., the sex of the head of household) and of the CSA (e.g., the age group, sex, marital status, and role in the community) were not significantly associated with birth reporting in the multivariate analysis.

### Factors associated with the likelihood of capturing deaths by CSAs.

Considering individual-level factors, adult deaths were more likely to be reported by the CSAs compared with infant deaths (for age 50 and above: aOR = 1.49; 95% CI = 1.10–2.02). Deaths of heads of household or their partners or their biological children were more likely to be reported than deaths of other household members (for head or partner: aOR = 1.69; 95% CI = 1.38–2.02).

Among cluster and CSA factors, deaths that occurred in smaller clusters were more likely to be reported than deaths in larger clusters (for < 1,000 people: aOR = 1.83; 95% CI = 1.32–2.55). Deaths in rural clusters were more likely to be reported than deaths of urban clusters (aOR = 1.41; 95% CI = 1.07–1.85). Deaths in clusters monitored by older CSAs were more likely to be reported than deaths in clusters monitored by younger CSAs (for 25- to 49-year-olds: aOR = 1.78; 95% CI = 1.29–2.46). Deaths in clusters monitored by less-educated CSAs were more likely to be reported than deaths in clusters monitored by higher-educated CSAs (aOR = 1.35; 95% CI = 1.03–1.76) (Table 4).

Overall, the reporting of deaths was significantly higher in most of the provinces than in Zambezia. However, in Niassa (aOR = 0.54; 95% CI = 0.29–0.98), Cabo Delgado (aOR = 0.61; 95% CI = 0.39–0.94), Nampula, and Sofala, the reporting of deaths was lower than or similar to Zambezia.

Characteristics of the deceased (e.g., sex), of the household (e.g., sex and age group of the head of household), and of the CSA (e.g., sex and role in the community) were not significantly associated with death reporting in the multivariate analysis.

## DISCUSSION

There was substantial underreporting of vital events from March 2018 to December 2019, with only 54% of births and 45% of deaths reported. Our study found a high correlation in the level of birth and death underreporting, even though we noted a 9-pp difference. Similar underreporting of deaths was found in the recently launched Indonesia SRS, which showed 55% of deaths were reported in 2016.^17^ A study conducted in two districts of Malawi concluded that the health surveillance agents (HSAs) reported in 2010 (January–December) about 57% of expected births and only 48% of expected under-five deaths; those rates declined from October 2010 to September 2011, with 50% of expected births and only 28% of expected under-five deaths reported.^21^^–^^23^

We found that reporting of births and deaths by community workers was affected by individual, household, and community characteristics.^42^^–^^46^ Because heads of households are the entry points to each household, events pertaining to them are more likely to be captured than events affecting other members of the households. We found that births of biological children of the head of household and deaths of heads of household and their partners were more likely to be reported than births and deaths of other family members.

Adult deaths were more likely to be reported than infant deaths in COMSA Mozambique. Similar results were found in other Asian SRSs. In Vietnam, about 58% of infant deaths were not reported, compared with only 16% of deaths among adults aged 50 and older.^47^ In China, the underreporting rate of children under 5 years of age was significantly higher than that of people 5 years old and above (35% and 17%, respectively).^11^^,^^24^^–^^26^ A multi-country study in sub-Saharan Africa on the validation of community vital events found similar underreporting rates among children under 5 years old. The HSAs in Malawi and the health extension workers (HEWs) in Ethiopia were more likely to report older deaths than younger deaths, with higher underreporting of neonatal deaths.^21^^–^^23^^,^^48^

Events in larger clusters were likely to be missed by CSAs in Mozambique. The multi-country study found higher reporting rates in Mali (90% of births and under-five deaths) with on average 412 people per community, as compared with Malawi (about 65% of births and 50% of under-five deaths) with about 1,000 people per community and Ethiopia (about 30% of births and 20% of under-five deaths) with about 5,000 people per community monitored by two HEWs.^21^^–^^23^ This raises a question about the size of a manageable cluster in the context of Mozambique. Mozambique’s national cadre of CHWs, known as “agentes polivalentes elementares,” serves a population of 2,500–5,000, which is about 500–1,000 households per community.^49^ However, their event reporting rates have yet to be assessed in Mozambique. Our analysis showed no effect of the role of CSA as a factor of underreporting in Mozambique. However, APEs missed 18% and 20% more births and deaths, respectively, than CSAs, after controlling for other factors, such as the type of cluster they work in (size, residence area, etc.) or CSA sex, age, education, etc. The APEs have other health-related assignments (health promotion and education, community case management) that take priority over reporting of vital events. Therefore, adding vital event registration as a task on top of other duties of CHWs may lead to poor event reporting and is not recommended. The design of COMSA has considered cluster sizes of approximately 300 households as manageable by a community worker. Although small clusters have a higher likelihood of capturing events, critical factors, such as the support and motivation of the CSAs (salary, transportation, communications, and close supervision), are paramount in ensuring high-level reporting.

Births and deaths in rural clusters were more likely to be reported than events in urban clusters. This can be explained by higher homogeneity and less mobility in the rural population compared with the urban population. Similar results were found in the mortality registration system in Indonesia, which found that the completeness of death reporting was higher in rural areas (73%) than in urban areas (52%).^16^ The China Disease Surveillance Points system, however, found a significantly higher underreporting of deaths in rural areas than in urban areas because deaths are directly reported in health facilities (hospitals, community health centers, and village clinics), and lower-level health facilities lack doctors to complete the death certificate and transfer the information.^24^^,^^26^

Community surveillance agents living outside their catchment areas have about 25% lower probability of reporting vital events compared with those who live in the community where they have been assigned to work. In the COMSA study, about half of CSAs live outside the community they are assigned to. Although residing in the cluster was an initial recruitment criterion, identifying an available literate community worker who was endorsed by the community was not straightforward, and many communities could not fulfill such a requirement. In the Malawi study, about the same proportion of HSAs lived outside their catchment areas, and about 35% of births and 50% of under-five deaths were not reported, as compared with the Mali study where all CHW (relais) lived in the catchment areas with a higher rate of reporting births and under-five deaths. Conversely, in Ethiopia, all HEWs lived in their catchment areas but missed about 70% of births and 80% of deaths. The Ethiopia model placed two HEWs at a fixed health post and tasked them to monitor a community of about 5,000 people.^21^^,^^23^

Community surveillance agents with a secondary or higher level of education were about 20% less likely to report vital events than CSAs with a primary level of education. This may be related to their community of residence. Only 43% of CSAs with a secondary education live in the community they are assigned to, as compared with 55% of CSAs with a primary education. Also, 33% of CSAs with a secondary education live in urban clusters, compared with 18% of CSA with a primary level. The low level of remuneration (US$25 per month) may also affect their availability to work on the project because many CSAs with a higher level of education may have other activities that generate more revenue compared with lesser-educated CSAs. Thus, they may allocate less time to the CSA work. About half of the CSAs with secondary level education are APEs or work at or a member of an organization. A multi-country study showed that less-educated volunteer CHWs (relais) in Mali, with no formal education, have reported about 90% of births and under-five deaths, compared with more educated HSAs in Malawi (i.e., with at least 10 years of schooling) who have reported about 65% of births and 50% of under-five deaths. In Ethiopia, HEWs with more than a tenth-grade education reported only 30% of births and 20% of under-five deaths.^21^^–^^23^

The province of residence had a significant effect on event reporting among CSAs. CSAs in Zambezia were less likely to report births and deaths than CSAs in other provinces. We found consistent combinations of birth and death underreporting in most provinces (Tete, Manica, Inhambane, Gaza, Maputo Province, and Maputo City). However, inconsistent high levels of underreporting for births and deaths were found in Niassa, Cabo Delgado, Nampula, and Sofala. Birth and death differences in adjusted odds ratios were large and the respective CIs do not overlap, meaning the difference between birth and death underreporting is statistically significant in those provinces. In sum, our analysis demonstrated a differential motivation in identifying births and deaths across the provinces. Zambezia, the province with the largest number of clusters, was also the province that performed worse in the identification and reporting of births. On the other hand, several provinces performed as poorly as or worse than Zambezia for death reporting. Although the reasons for the differential motivation are unclear, the patterns observed may be due to community workers in Zambezia prioritizing the identification of deaths more than births. Babies born alive were more likely to be reported compared with stillbirths. Underreporting of stillbirths is common in many other settings, particularly in LMICs; it has been estimated that household survey data underreport stillbirths by 36% and vital registration by 19–30%.^42^ Underreporting of stillbirths can be related to reluctance to ask about babies that are born dead,^43^ the openness of families to disclose stillbirths,^44^ or the stigma associated with stillbirths.^45^^,^^46^ COMSA tools include registration of pregnancies. CSAs were trained to write down the expected date of childbirth and then revisit the family within two weeks after the delivery. However, COMSA has not set up an electronic reminder to the CSA, and therefore CSAs may not systematically follow up on reported pregnancies. COMSA implementers should pay more attention to maintaining pregnancy cohort registers and assess the best strategies to remind CSAs of expected dates of deliveries. The close follow-up of pregnancies could improve timely and accurate recording of births, stillbirths, and early neonatal deaths as well as maternal deaths.

There was no effect of the sex of the baby or the deceased as a factor of reporting in Mozambique. About 53% of male babies and 54% of female babies were reported by the CSAs. Similar results were found in Ethiopia, where similar proportions of male and female babies were reported by the HEWs. Those marginal sex differences in the birth reporting were then confirmed during the validation household study in Ethiopia.^48^ However, several studies in other contexts found that male babies are more likely to be reported than female babies.^21^^,^^23^ About 45% of male deaths and 44% of female deaths were reported by the CSAs. Many studies have found little sex differences in death reporting. The Indonesian SRS has reported 51% of female deaths and 55% of male deaths.^17^ The differences in reporting among male and female deaths between the COMSA and other Asian SRS were comparable, with about 81% of female deaths and 81% of male deaths reported in Vietnam 2009^50^ and 86% of female deaths and 88% of male deaths reported in China in 2009.^11^ However, the death reporting rates were much higher in Asian systems than the Mozambican system.

Our findings show the importance of conducting an annual survey of the population and recent vital events to complete community-based birth and death reporting to correct annual estimates. This survey can also guide strengthening the surveillance system to improve reporting by community workers on a regular basis. In the context of COMSA, additional field procedures were implemented to increase the completeness of vital events reporting. Each CSA was paired with two or three key community informants to report events on a regular basis, so the CSA could visit specific households to collect the information. Then, CSAs were tasked to visit every household at least once a quarter to ask about events they may have missed during that period. Also, a supervision system has been put in place to ensure that each CSA is visited a least once every quarter by the supervisor (VASA interviewer) and that communication continues with at least monthly phone calls or *Whatsapp* messages. Additional steps should be taken to ensure that standard protocols throughout the supervision and reporting system in place are followed by all entities and adapted to the context if necessary. Strengthening the supervision system could improve community-based events reporting.^20^ A multi-country study including Mozambique showed that strengthening the reporting capacity of community health workers and supervisors has significantly improved the integrated community case management data quality and reporting.^51^

### Limitations.

Our work has several important limitations. First, the reference dataset we used to assess CSA completeness data is far from perfect. Household surveys collecting births and deaths have been reported to not capture all vital events due to recall error, misreporting, and interviewer error.^52^ However, such periodic surveys are commonly used to correct estimates from community surveillance.^21^^,^^53^

In our study, considerations were taken to collect high-quality data on vital events. Assessment interviewers were provided with much information related to the household identification (household number and name of the head of household); the names of household members; and, more importantly, all births and deaths reported by the surveillance with events’ identifying information such as year of birth or year of death, sex of the baby or the deceased, age of the deceased, and name of the parents (if any). Therefore, the assessment did identify substantially more vital events than the surveillance. Thus, we can consider our reference data are more complete than a regular household survey.

Second, the matching processes we used in this study may bias the results because we relied on interviewers to correctly match the events reported by the surveillance and the assessment data. The national identification number on birth certificates and the national identification card were not used in this study because many people in Mozambique do not have such documents in rural settings. For instance, the 2017 housing and population census data revealed that only one-third of babies born 12 months prior to the census were registered in the CRVS, with 36% in urban and 31% in rural areas (calculations from authors; data not shown). However, we have conducted a second layer of data matching using statistical methods to complete this field process.

Third, our completeness of birth and death data, as the proportion of recorded births and deaths by the surveillance out of the total births and deaths recorded by the assessment, is based on one-way matching. Using the capture-recapture method, however, we found that the assessment data missed about 2% of deaths and 8% of births reported by the surveillance (data not shown). Therefore, we can consider that the completeness of the assessment data was about 87% for deaths and 95% for births. Additional analysis is ongoing to better understand the characteristics of vital events more likely to be missed during a special survey in the context of COMSA to improve data collection and produce more accurate annual estimates.

## CONCLUSION

In LMICs, many deaths and births still occur outside the health facility, and most countries do not have a functioning CRVS system that allows accurate measurement of mortality. A strong community-based birth and death reporting system can improve the identification of such vital events to monitor progress toward SDGs. However, similar to previous studies, sole reliance on community workers results in substantial under-capture of vital events, which can lead to severe underestimates of vital rates. Several factors related to individuals, their relationships to the head of household, and the cluster size are significantly correlated with birth and death reporting. The findings support the importance of conducting annual updates of population listings and vital events to complete the community surveillance data to produce accurate annual estimates. First, a fully functioning SRS must adopt a dual system with high-quality surveys or other ways to estimate underreporting periodically. Such field procedures are conducted in Asian SRSs. In Bangladesh, a yearly population census and 3-month household vital events surveys are conducted by supervisors.^54^ In India, half-yearly complete household surveys are conducted by an independent team of surveyors.^55^ Second, measures to improve community-based events reporting may include reducing large size clusters into a more manageable work area by a community worker and paying special attention to urban clusters because the under reporting is larger, which may require even a smaller cluster size. Finally, other considerations are 1) strengthening the supervision of the community workers to ensure quarterly routine visits of all households by community workers are systematically conducted, 2) continuous training of community workers is in place to emphasize the importance of asking questions about all members in the household, and 3) strong and continuous support and motivation of community workers, who form the backbone of any community surveillance, are also critical for producing quality data and sustaining the surveillance system.

## Financial Disclosure

Financial support: This study was funded by the Bill & Melinda Gates Foundation (grant number OPP1163221).
